# Overestimated discriminatory power of MALDI-TOF mass spectrometry for typing of carbapenem-resistant *Klebsiella pneumoniae* clones

**DOI:** 10.1017/S0950268819002097

**Published:** 2019-12-17

**Authors:** Fei Jiang, Ziyan Kong, Chen Cheng, Haiquan Kang, Bing Gu, Ping Ma

**Affiliations:** 1 Department of Laboratory Medicine, the Affiliated Hospital of Xuzhou Medical University, Xuzhou, Jiangsu Province, China; 2 School of Medical Technology, Xuzhou Medical University, Xuzhou, Jiangsu Province, China

**Keywords:** Bacterial typing, *Klebsiella pneumoniae*, MALDI-TOF MS, MLST, PFGE

## Abstract

Homology surveillance of carbapenem-resistant *Klebsiella pneumoniae* (CRKP) is critical to monitor and prevent outbreaks of nosocomial infections. In the present study, a matrix-assisted laser desorption/ionisation-time of flight (MALDI-TOF MS)-based method was evaluated as a rapid tool for typing CRKP in comparison with pulsed-field gel electrophoresis (PFGE) and multi locus sequence typing (MLST). Drug-resistant phenotypes and genotypes of 44 CRKP isolates were detected by microdilution broth method and polymerase chain reaction, and typed by PFGE, MLST and MALDI-TOF MS. Simpson's Index of Diversity was used to evaluate taxonomic diversity, Adjusted Rand Index (ARI) for congruence between the typing methods and Wallace coefficients (*W*) for the ability of either method to predict each other. Forty-four CRKP isolates of 15 sequence types (STs) produced either *NDM-1* (*n* = 16), *NDM-5* (*n* = 9) or *KPC-2* (*n* = 19) carbapenemases. PFGE differentiated these isolates into 16 distinct types, and two deoxyribonucleic acid profiles were assigned to ST337 and ST11, respectively. MALDI-TOF MS failed to clearly delineate between clusters on dendrograms based on principal components analysis and main spectrum profile. The chosen parameters resulted in a maximum ARI of 0.310 (95% CI 0.088–0.531) between MALDI-TOF MS typing and the PFGE reference, indicating a low ability of the former to correctly identify related isolates. Likewise, the maximum *W* coefficient of 0.367 (95% CI 0.203–0.532) showed that MALDI-TOF MS had a lower predictive power than PFGE. We conclude that MALDI-TOF MS lacks the discriminatory power necessary for clone assignment of CRKP isolates and consequently cannot be considered as a rapid and creditable method for this purpose.

## Introduction

*Klebsiella pneumoniae* is one of the most common pathogens associated with community-acquired and hospital-acquired infections. In recent years, the mortality in patients with carbapenem-resistant *K. pneumoniae* (CRKP) infection has been increasing [1 2]. According to national bacterial resistance monitoring data of China Antimicrobial Surveillance Network, the detection rate of CRKP increased from 5% in 2008 to 25% in 2018. In the meantime, the increasing number of nosocomial outbreaks due to CRKP worldwide has presented great challenges for clinical treatment, prevention and control of such infections [3–6].

Currently, pulsed-field gel electrophoresis (PFGE) and multilocus sequence typing (MLST) are widely used techniques for molecular typing of bacteria. PFGE offers a high level of intraspecies resolution with good reproducibility while MLST has the advantages of standardisation of databases which allows parallel comparisons of data from different regions to facilitate global tracking of bacterial clones. Although whole genome sequencing is increasingly replacing PFGE and MLST as a gold standard for molecular typing of bacteria [7], it is, in keeping with the foregoing techniques, time-consuming and expensive and not suitable for real-time surveillance of nosocomial infections.

With the almost routine application of matrix-assisted laser desorption/ionisation-time of flight (MALDI-TOF) in the clinical microbiology laboratory for species identification, wider exploration of additional generated data has been made regarding its suitability for the identification of antimicrobial resistance markers [8] and strain typing [9]. We therefore undertook a study to (i) evaluate the capability of MALDI-TOF MS as a rapid and accurate typing method compared with PFGE and MLST for CRKP isolates and (ii) describe the microbiologic characteristics (e.g. resistance phenotype and associated resistance genes) of these isolates.

## Materials and methods

### Bacterial isolation and sample preparation

A total of 44 non-duplicate *K. pneumoniae* isolates were collected from three hospitals in Northern Jiangsu province, China between 2016 and 2018. The isolates were recovered from sputum (*n* = 28), blood (*n* = 6), urine (*n* = 6), pus (*n* = 2), cerebrospinal fluid (*n* = 1) and bronchial perfusate (*n* = 1). All were identified to species level (score >2.0) using MALDI-TOF MS (Bruker Daltonics, Bremen, Germany). Isolates were screened for susceptibility to imipenem and meropenem in accordance with Clinical and Laboratory Standards Institute criteria. Isolates were stored at −80 °C, and cultured on Columbia sheep blood agar plates (KeMaJia, Shanghai, China) for 24 h at 37 °C.

### Detection of carbapenem resistance genes

The 50 µl reaction mixture contained the following: 25 µl 2 × Taq Master Mix, 3 µl template deoxyribonucleic acid (DNA), 2 µl 10 µmol/l of each primer and 18 µl ddH_2_O. Each polymerase chain reaction (PCR) involved an initial denaturation at 94 °C for 5 min, followed by 30 amplification cycles each consisting of a denaturation at 94 °C for 45 s followed by annealing at 56 °C for 45 s and an extension at 72 °C for 45 s. Final extension was carried out at 72 °C for 10 min. The primer sequences and temperature of PCR annealing are listed in Table 1. Primer synthesis and amplified product sequencing were carried out by the GenScript Company (Nanjing, China).

**Table 1.** Primers sequencing of resistance genes and seven house-keeping genes for *K. pneumoniae*

Genes	Primers sequencing (5′−3′)	Annealing temperature (°C)
Resistance genes		
*KPC*	F: ATGTCACTGTATCGCCGTC	58
	R: TTACTGCCCGTTGACGCC	
*NDM*	F: GAAGCTGAGCACCGCATTAG	58
	R: GGGCCGTATGAGTATTGC	
House-keeping genes		
*rpoB*	F: GGCGAAATGGCWGAGAACCA	50
	R: GAGTCTTCGAAGTTGTAACC	
*gapA*	F: TGAAATATGACTCCACTCACGG	60
	R: CTTCAGAAGCGGCTTTGATGGCTT	
*mdh*	F: CCCAACTCGCTTCAGGTTCAG	50
	R: CCGTTTTTCCCCAGCAGCAG	
*pgi*	F:GAGAAAAACCTGCCTGTACTGCTGGC	50
	R: CGCGCCACGCTTTATAGCGGTTAAT	
*pho*	F: ACCTACCGCAACACCGACTTCTTCGG	50
	R: TGATCAGAACTGGTAGGTGAT	
*infB*	F: CTCGCTGCTGGACTATATTCG	50
	R: CGCTTTCAGCTCAAGAACTTC	
*tonB*	F: CTTTATACCTCGGTACATCAGGTT	45
	R: ATTCGCCGGCTGRGCRGAGAG	

### Pulsed-field gel electrophoresis (PFGE)

PFGE with dendrogram analysis was performed as previously described [10]. *K. pneumoniae* agarose plugs were digested with protease K and restriction enzyme *XbaI* (Takara, Dalian, China). *Salmonella enterica* serovar Braenderup strain H9812 restricted with *XbaI* was used for molecular weight determinations. DNA fragments were separated using a CHEF-DR II PFGE system (Bio-Rad, Hercules, CA, USA) for 20 h at 6 V/cm and 14 °C, with a pulse angle of 120° and pulse times from 4–40 s. Strains were considered as the same PFGE type if they possessed >80% pattern similarity.

### Multi locus sequence typing (MLST)

MLST was performed as reported by Diancourt *et al*., [11] (http://bigsdb.pasteur.fr/klebsiella/primers_used.html). The 50 µl reaction mixture contained the following: 25 µl 2 × Taq Master Mix, 3 µl template DNA, 2 µl 10 µmol/l of each primer and 18 µl ddH_2_O. Each PCR involved an initial denaturation at 94 °C for 5 min, followed by 30 amplification cycles each consisting of a denaturation at 94 °C for 45 s followed by annealing at 50 °C except for *gapA* (60 °C) and *tonB* (45 °C) for 45 s and an extension at 72 °C for 45 s. Final extension was carried out at 72 °C for 10 min. The primer sequences and temperature of PCR annealing are listed in Table 1.

### MALDI-TOF MS

A single colony of fresh overnight cultures was transferred to a stainless steel target using a sterile toothpick and overlaid with 1 µl 70% formic acid and after drying in room temperature, 1 µl matrix solution containing acetonitrile, water and trifluoroacetic acid (50 : 47.5 : 2.5) was added to each target spot.

Bruker MALDI Microflex LT with a linear positive mode of spectra acquisition, at a laser frequency of 60 Hz was used for the measurements. A broad *m/z* range (2–20 kDa) of spectra was automatically acquired using the AutoXecute acquisition control software (Flex control 3.0, Bruker Daltonics, Leipzig, Germany) with the following instrument settings: ion source 1 at 19.96 kV, source 2 at 18.06 kV, and lens at 6 kV, 240 laser shots/spot in 40 shot steps. A bacterial test standard was used for calibration and validation, and analysis was performed as recommended by the manufacturer for species identification.

The mass spectra of each quadruplicate of the respective isolates with a score value of >2.0 were considered for dendrogram preparation. Preprocessing of the spectra (baseline subtraction, smoothing, and noise calculation) was performed using the default settings, before summarising the spectra of technical and biological replicates, to yield one summary spectrum per isolate. Principal components analysis (PCA) and main spectrum profile (MSP) dendrograms were generated by using the respective functionality of the MALDI Biotyper 3.1 offline client. The spectra of all isolates tested were analysed by a score-oriented dendrogram using desired distance level as the cutoff.

### Data analysis

Simpson's Index of Diversity (DI), Adjusted Rand Index (ARI) and Wallace coefficients (*W*) were calculated using the online tool at www.comparingpartitions.info. A DI = 1.0 signifies that the typing method distinguishes between all isolates, whereas a DI = 0 indicates homogeneity of isolate type. The ARI quantifies the degree of congruence of the resulting cluster composition and a value of 1.0 signals complete concordance between two typing methods. However, the *W* coefficient provides a finer comparison between two typing methods, since the value indicates the probability that two isolates assigned to the same type by one method are also classified in the same type by the other method. A high *W* coefficient (values close to 1) indicates that partitions defined by a given method could have been predicted from the results of another method [12].

## Results

### Antimicrobial susceptibility testing and carbapenems resistance gene

All 44 isolates exhibited resistance to imipenem (4 to ⩾16 mg/l) and meropenem (4 to ⩾16 mg/l), among which 16 carried the *NDM-1* gene, and nine carried the *NDM-5* gene; 19 isolates were positive for the *KPC-2* gene.

### Typing by PFGE and MLST

**Fig. 1.**
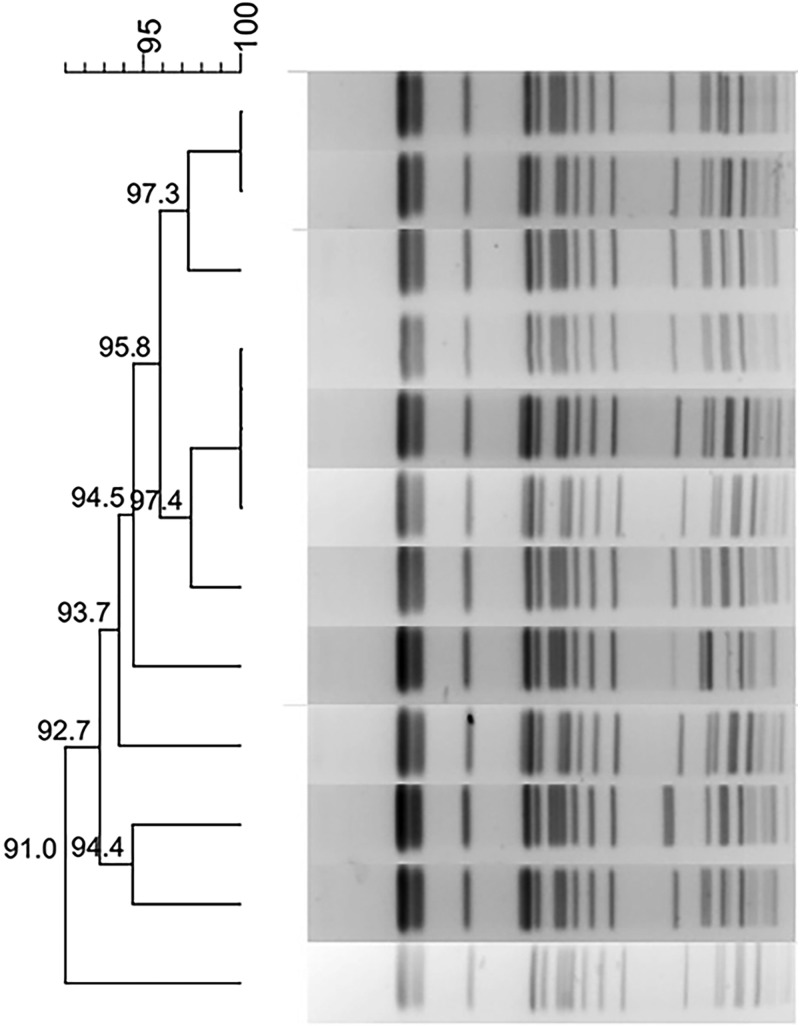
Typing results of PFGE and MLST from 44 CRKP isolates. Two PFGE clones belong to ST337 and ST11, respectively.

Phylogenetic trees derived from the PFGE analysis separated all 44 CRKP isolates into 16 clusters (PC1–PC16) according to 80% similarity while MLST differentiated all isolates into 15 clusters. Isolates of the two prevalent PFGE clusters fell into ST337 (*n* = 14) and ST11 (*n* = 15), respectively. The distribution of other isolates was ST25 (*n* = 2), ST37 (*n* = 2), and one isolate each in 10 other STs (Fig. 1).

### MALDI-TOF MS-based typing

**Fig. 2.**
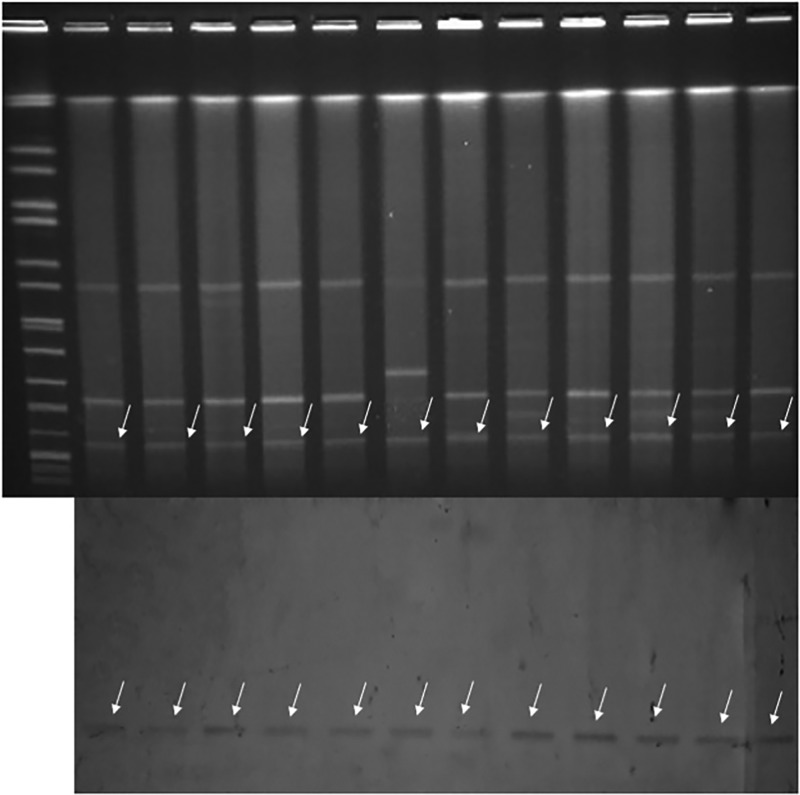
Three-dimensional plot generated by MALDI-TOF MS. Four PCA clusters were identified. Strains from distinct PFGE clusters (PC) were grouped into one MALDI-TOF cluster, and strains belonging to the same PFGE cluster were assigned to more than one MALDI-TOF clusters.

Based on the complete mass spectra, a PCA revealed that all isolates were divided into four clusters using an arbitrary distance level as the cutoff, as described previously [13 14]. Isolates from distinct PFGE and MLST clusters were grouped together in a single MALDI-TOF cluster, and those grouped in the same PFGE and MLST cluster were assigned to more than one MALDI-TOF cluster (Fig. 2). By Simpson's DI scores, MALDI-TOF MS (DI = 0.614, 95% CI 0.522–0.707) was less discriminating than PFGE (DI = 0.805, 95% CI 0.721–0.890) and MLST (DI = 0.791, 95% CI 0.704–0.877).

Using PFGE as the reference, the chosen parameters resulted in a maximum ARI of 0.310 (95% CI 0.088–0.531), which indicated an extremely low ability of MALDI-TOF MS to identify related isolates. The *W* coefficient between PFGE and MALDI-TOF MS typing (*W*_PFGE→MALDI-TOF_ = 0.728, 95% CI 0.529–0.927) was much higher than the expected value under independence (0.386), but trying to predict MALDI-TOF MS type from a PFGE type would lead to error rates of 7.3% to 47.1% among strain pairs with the same PFGE type. However, the reverse comparison (*W*_MALDI-TOF→PFGE_ = 0.367, 95% CI 0.203–0.532) leads to much higher error rates (from 46.8% to 79.7%). The congruence between PFGE and MLST gave an ARI value of 0.954 (95% CI 0.862–1.000). Similarly, using PFGE to predict MLST, and reverse, the *W* coefficients were 1.000 (95% CI 1.000–1.000) and 0.929 (95% CI 0.810–1.000) respectively, which indicated good predictive power. It was noted that two *K. pneumoniae* clones (ST337 and ST11) were grouped into two and six MALDI-TOF clusters respectively (ARI = 0). For the other epidemiologically unrelated isolates, the ARI was 0.035 (95% CI 0–0.131) between PFGE and MALDI-TOF when using an arbitrary distance level of 1000 as the cut-off.

A similarity cut-off value was chosen that lead to the generation of several clusters. The DI, ARI and *W* coefficient were then calculated to quantify the diversity of MALDI-TOF MS and congruence between MALDI-TOF MS typing and the PFGE. Different clustering cut-off values resulted in a wide range of cluster numbers (DI, 0.566–0.747), but the ARI (0.121–0.310) still showed low congruence between MALDI-TOF MS and PFGE. The ability of MALDI-TOF typing to predict PFGE typing (*W*_MALDI-TOF→PFGE_) remained at a lower level (0.273–0.367).

The UPGMA algorithm used for clustering and the dendrogram based on MSP analysis clearly separated members of the same *K. pneumoniae* lineage into different phylogroups. The corresponding ADI between MALDI-TOF MS and PFGE and MLST was maximal, based on three phylogroups, giving values of 0.192 (95% CI 0–0.404) and 0.175 (95% CI 0–0.386) respectively. Nevertheless, the algorithm could not completely distinguish between two clones from the others and this implied a low ability of MALDI-TOF MS to cluster related isolates.

## Discussion

Rapid and accurate strain level subtyping is necessary to highlight genotypic differences among multidrug-resistant isolates and to trace the sources and distribution of clones in outbreaks of nosocomial infections, or extended epidemiological investigations. This study was performed to determine the clonal distributions among 44 CRKP isolates and identify the presence of *NDM* and *KPC* genes conferring high level resistance to carbapenems. We aimed to compare the discriminatory power of MALDI TOF MS and its concordance with clonal designations, determined by PFGE and MLST techniques, for the collection of isolates.

A high degree of congruency was found between the results of PFGE and MLST typing (ARI 0.954), and the *W* coefficient value indicated that strains classified in the same cluster by PFGE were also grouped in the same MLST clusters. However, contrary to our expectation, there was low congruence between MALDI-TOF MS and PFGE typing (ARI_max_ = 0.310), which implied that it is difficult to obtain a similar level of strain discrimination using MALDI-TOF MS compared with the PFGE reference method. The observation that PFGE type could predict MALD-TOF MS type to a greater extent than the reverse is supported by the non-overlapping 95% confidence intervals of the *W* coefficient (0.728 (0.529–0.927) *vs.* 0.367 (0.203–0.532)). However, the former was still associated with a high (47.1%) error rate, which is not credible for practical use.

Several attempts have been made to apply MALDI-TOF MS to resolve between strains of *K. pneumoniae*, but with different conclusions [15–19]. Two groups [15 16] reported that MALDI-TOF MS was a promising tool for this purpose but the low number of isolates (10 of 6 PFGE types) in one study [15] and the lack of calculation of the congruence between MALDI-TOF MS and other reference methods (WGS, PFGE or MLST) [16] limits firm conclusions to be made. The other three studies [17–19] found MALDI-TOF-based typing to be less discriminatory than whole genome sequencing, PFGE or MLST for typing *K. pneumoniae*. The limitations of MALDI-TOF-based typing were further pointed out by Spinali *et al*., [20] and Sauget *et al*., [21] who attributed its shortcomings as a typing tool to technological and biological factors such as strain sets, culture conditions, definition of specific MS peaks, statistical analysis, etc. The choice of cut-off similarity values for clustering in order to allow valid comparisons of strain-specific data was also considered to be crucial [20]. In this study, we applied strictly the same conditions to our samples to reduce as far as possible technical variation and then compared different cut-off values to differentiate clusters of MALDI-TOF MS. However, this did not result in a substantial increase in the ARI value leading us to conclude that MALDI-TOF MS did not provide the required resolution for type identification of our CRKP isolates.

In order to define an epidemiological link between two strains by PFGE, depending on the species, up to three band differences in a profile of 20 to 30 DNA fragments are generally allowed PFGE [22]. As MS data produce an average of 100 peaks in a profile, Spinali *et al*., [20] proposed that an average difference of 15 peaks could be accepted in MALDI-TOF typing as the discriminatory cut-off between epidemiologically unrelated isolates. Likewise these authors introduced the concept of using ratios of the number of variable MS peaks to the number of PFGE restriction fragments, to inform differentiation guidelines for different species, and concluded MALDI-TOF MS showed potential as a rapid and inexpensive strain typing tool. This conclusion is not corroborated by our findings which unequivocally show that MALDI-TOF MS is a poor tool for real-time homology surveillance of *K. pneumoniae* based on several indices of clustering concordance. Further ongoing analysis in our laboratory of duplicate strains and a larger dataset appears to confirm our conclusion that MALDI-TOF MS currently lacks the discriminatory power and congruence values with other methods that would be required of a methodology for epidemiological surveillance of antimicrobial resistant *K. pneumoniae* in the hospital setting.
