# Overestimated discriminatory power of MALDI-TOF mass spectrometry for typing of carbapenem-resistant *Klebsiella pneumonia* clones – CORRIGENDUM

**DOI:** 10.1017/S0950268820000035

**Published:** 2020-01-20

**Authors:** Fei Jiang, Ziyan Kong, Chen Cheng, Haiquan Kang, Bing Gu, Ping Ma

**Original:**

**Fig. 1.**
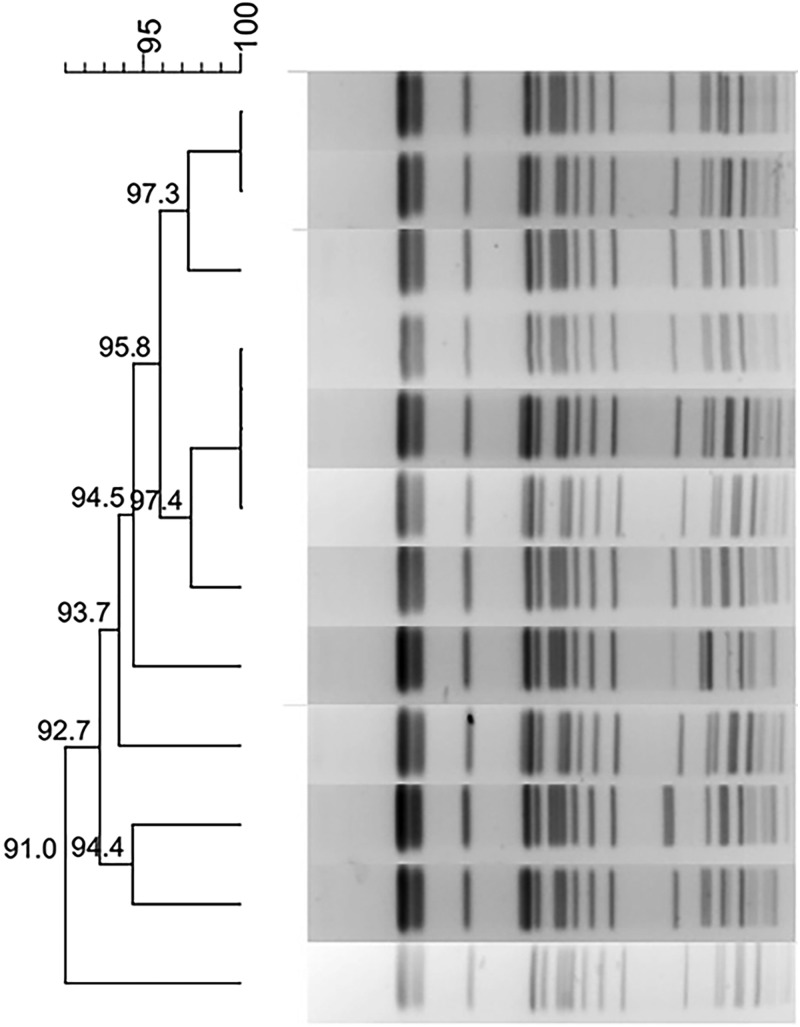
Typing results of PFGE and MLST from 44 CRKP isolates. Two PFGE clones belong to ST337 and ST11, respectively.

**Correction:**

**Fig. 1.**
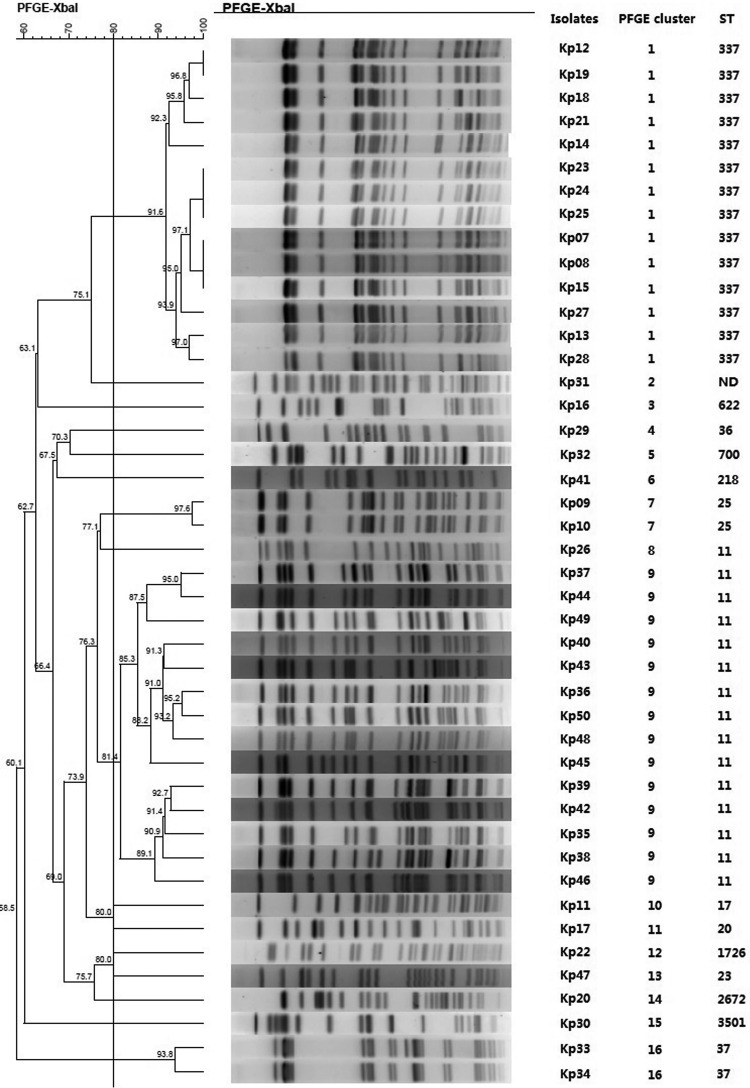
Typing results of PFGE and MLST from 44 CRKP isolates. Two PFGE clones belong to ST337 and ST11, respectively.

**Original:**

**Fig. 2.**
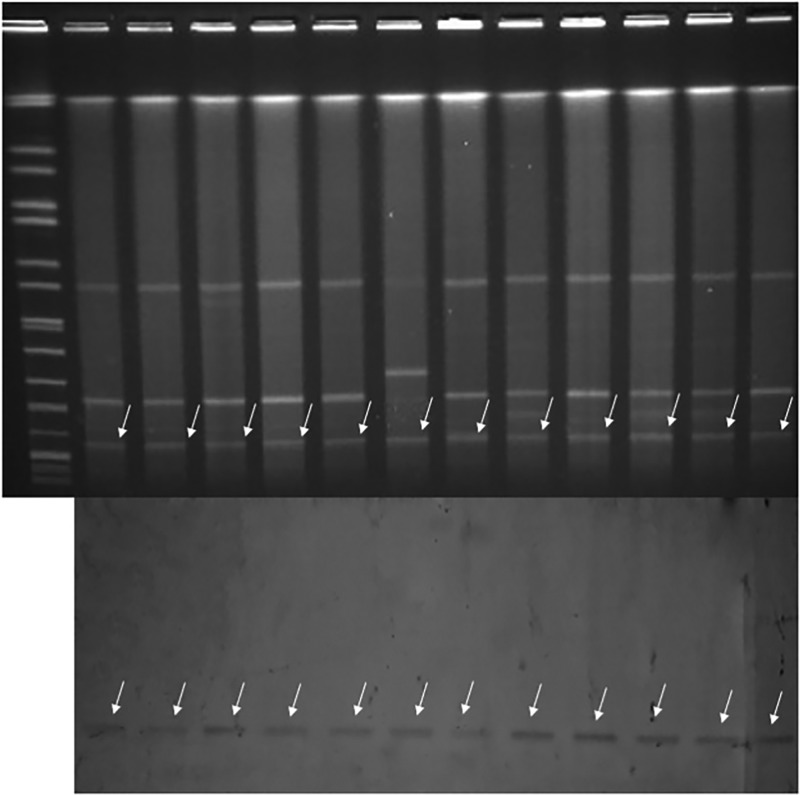
Three-dimensional plot generated by MALDI-TOF MS. Four PCA clusters were identified. Strains from distinct PFGE clusters (PC) were grouped into one MALDI-TOF cluster, and strains belonging to the same PFGE cluster were assigned to more than one MALDI-TOF clusters.

**Correction:**

**Fig. 2.**
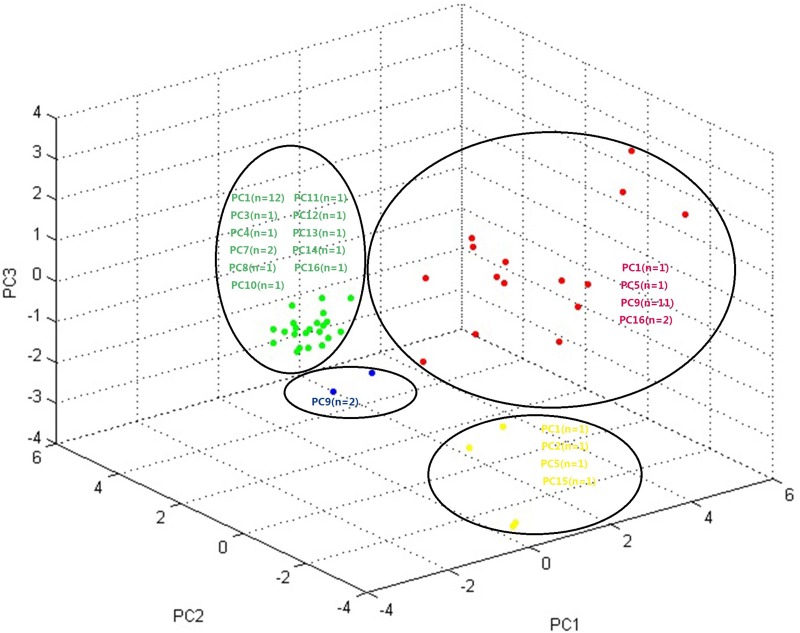
Three-dimensional plot generated by MALDI-TOF MS. Four PCA clusters were identified. Strains from distinct PFGE clusters (PC) were grouped into one MALDI-TOF cluster, and strains belonging to the same PFGE cluster were assigned to more than one MALDI-TOF clusters.
